# Code-Free Automated Machine Learning for Referable Glaucoma Classification From Retinal Fundus Photographs: A Proof-of-Concept Study Using Google Cloud Vertex AI

**DOI:** 10.7759/cureus.115841

**Published:** 2026-09-05

**Authors:** Mark A Bachir, Alexander Bachir, Neel Nawathey, Akshay J Reddy, Jasmeen K Cheema, Telak Brahmbhatt, Jacob Webster, Rakesh Patel

**Affiliations:** 1 Internal Medicine, California Northstate University College of Medicine, Elk Grove, USA; 2 Medicine, California Health Sciences University, Fresno, USA; 3 Osteopathic Medicine, Touro University California, Mare Island, USA; 4 Medicine, California University of Science and Medicine, Colton, USA; 5 Biomedical Sciences, University of California Davis, Davis, USA; 6 Medicine, Lincoln Memorial University-DeBusk College of Osteopathic Medicine, Knoxville, USA; 7 Medicine, The University of Oklahoma, Norman, USA; 8 Internal Medicine, East Tennessee State University Quillen College of Medicine, Johnson City, USA

**Keywords:** artificial intelligence, automated machine learning, automl, deep learning, glaucoma, glaucoma screening, google cloud vertex ai, image classification, ophthalmic imaging, retinal fundus photography

## Abstract

Artificial intelligence has demonstrated substantial potential for automated glaucoma screening from retinal fundus photographs; however, conventional deep-learning development often requires programming expertise, specialized knowledge of neural-network design, and substantial computational resources. This study evaluated whether a commercially available, code-free automated machine-learning (AutoML) platform could generate a high-performing classifier for distinguishing referable glaucoma (RG) from non-referable glaucoma (NRG). A publicly available balanced retinal fundus image dataset derived from the EyePACS Artificial Intelligence for Robust Glaucoma Screening dataset was used. The study included 9,540 color fundus photographs, with 4,770 images in each class. Images were assigned to training (n=7,632; 80%), validation (n=954; 10%), and held-out internal testing (n=954; 10%) partitions. A single-label image-classification model was developed using Google Cloud Vertex AI AutoML (Google LLC, California, US) through its graphical interface without investigator-written machine-learning code or manual neural-network architecture design. Model training used a maximum computational budget of eight node-hours. Performance was evaluated using average precision, precision, recall, precision-recall analysis, confidence-threshold analysis, and a row-normalized confusion matrix. The AutoML model achieved an overall average precision of 0.974. At the default confidence threshold of 0.50, overall precision and recall were both 92.5%. Class-specific average precision was 0.977 for both RG and NRG. The model correctly classified 94% of NRG images and 91% of RG images. Deployment to a cloud-based prediction endpoint successfully generated class predictions and confidence scores for representative retinal fundus photographs. A fully graphical AutoML workflow therefore produced strong internal classification performance for distinguishing RG from NRG without investigator-written machine-learning code or manual neural-network development. These findings support the feasibility of commercial AutoML platforms as accessible tools for ophthalmic image-classification research. Because the model was evaluated using a single derived dataset without external testing, further validation across independent populations, institutions, and imaging systems is necessary before clinical implementation.

## Introduction

Glaucoma is a leading cause of irreversible vision loss worldwide and encompasses a heterogeneous group of optic neuropathies characterized by progressive damage to retinal ganglion cells and the optic nerve. Because structural and functional injury may develop before patients recognize visual symptoms, glaucoma can remain undiagnosed until permanent visual impairment has occurred. The global burden of glaucoma is expected to increase substantially with population aging, emphasizing the importance of timely detection and treatment. Early diagnosis and appropriate therapy remain central to slowing disease progression and preserving vision [1-3].

Color fundus photography provides a noninvasive method for evaluating the optic nerve head and surrounding retinal structures and is commonly incorporated into ophthalmic assessment and screening workflows. Interpretation of fundus photographs, however, depends on clinical expertise and may vary according to image quality, disease severity, and observer experience. Artificial intelligence (AI), particularly deep learning, has emerged as a promising approach for automatically identifying imaging features associated with ocular disease and generating reproducible classification predictions [4-7].

Multiple investigations have demonstrated that deep learning systems can identify glaucomatous or referable optic nerve abnormalities in retinal fundus photographs with high discriminative performance [8-11]. Studies using independent datasets and different imaging systems have further illustrated both the potential and the challenges associated with generalizing glaucoma classifiers beyond their development data [12,13]. More broadly, successful implementation of ophthalmic AI requires careful consideration of dataset quality, technical robustness, external validation, clinical integration, and appropriate interpretation of algorithmic predictions [14]. Increasing attention has also been directed toward explainable AI approaches intended to improve model transparency and facilitate clinically meaningful interpretation of automated predictions [15].

Despite these advances, conventional deep-learning development frequently requires expertise in programming, neural-network architecture selection, model optimization, and computational infrastructure. Automated machine learning (AutoML) offers an alternative approach by automating substantial portions of model development and allowing users to train image classifiers through graphical interfaces. Previous studies have demonstrated the feasibility of code-free or AutoML-based medical image classification across multiple imaging modalities [16-19]. These platforms may therefore broaden access to exploratory AI research among clinicians and biomedical investigators who do not have formal machine-learning programming expertise.

The objective of this proof-of-concept study was to evaluate the classification performance of Google Cloud Vertex AI AutoML (Google LLC, California, US) for distinguishing referable glaucoma (RG) from non-referable glaucoma (NRG) using retinal fundus photographs through a workflow requiring no investigator-written machine-learning code or manual neural-network architecture development. The primary purpose was not to establish a clinically deployable glaucoma diagnostic system or demonstrate superiority over custom deep-learning architectures. Rather, this study evaluated the feasibility and internal classification performance of an accessible, commercially available AutoML workflow for ophthalmic image-classification research.

## Materials and methods

Study design

This proof-of-concept model-development study evaluated a code-free workflow for binary classification of retinal fundus photographs using Google Cloud Vertex AI AutoML. Investigator-directed steps included uploading and importing the dataset, supplying the image labels and partition assignments through the import workflow, selecting the single-label image-classification task, specifying the training region and computational budget, initiating model training, reviewing the platform-generated evaluation results, and deploying the trained model. Image preprocessing and label assignment had been completed by the dataset developers before public distribution and were not independently modified by the investigators. Vertex AI AutoML automated feature extraction, neural-network architecture selection, optimization, and hyperparameter tuning. The study was reported with consideration of the Checklist for Artificial Intelligence in Medical Imaging (CLAIM) and TRIPOD+AI recommendations, where applicable [20,21]. The held-out test subset originated from the same overall dataset used for model development and was therefore considered an internal test set rather than an external validation cohort. Accordingly, the present analysis was designed to assess technical feasibility and internal classification performance rather than establish generalizability to independent clinical populations.

Dataset and reference standard

The study used EyePACS-AIROGS-light-V2, a publicly available retinal fundus image dataset distributed through Kaggle and derived from the Rotterdam EyePACS Artificial Intelligence for Robust Glaucoma Screening (AIROGS) dataset [22,23]. The original AIROGS resource contains more than 100,000 color fundus photographs obtained from a large and heterogeneous glaucoma-screening population [22].

The dataset used for the present experiment contained 9,540 retinal fundus photographs, including 4,770 labeled as RG and 4,770 labeled as NRG. For the Vertex AI experiment, 7,632 images were assigned to the training partition, 954 to the validation partition, and 954 to the held-out internal test partition, corresponding to an 80% / 10% / 10% distribution. Partitioning was performed at the image level rather than at the patient or eye level. No additional patient-level or eye-level grouping was applied by the investigators during partitioning. Partition assignments were encoded in the dataset import manifest and maintained throughout model development and evaluation.

The binary class labels supplied with the publicly available dataset were used as the reference standard. The investigators did not independently review, regrade, or relabel the fundus photographs. Accordingly, model performance represents agreement with the dataset-provided RG and NRG labels rather than an independently adjudicated glaucoma diagnosis.

Image preprocessing

No additional image preprocessing, manual image enhancement, optic-disc segmentation, or investigator-directed normalization was performed before model import. Images were used in the standardized format supplied with the derivative dataset. According to the dataset documentation, preprocessing performed before public distribution included identification and cropping of the retinal foreground, removal of excess black background, centering of the retinal field, resizing to 512 × 512 pixels, and application of a foreground mask to standardize image appearance [23]. The investigators did not independently assess the contribution of these preprocessing steps to model performance. Consequently, the reported performance reflects both the standardized preprocessing applied by the dataset developer and the subsequent AutoML classification workflow.

Google Cloud Vertex AI AutoML workflow

The retinal fundus images were uploaded to Google Cloud Storage and imported into Google Cloud Vertex AI as a single-label image-classification dataset. An import manifest associated each image with its Cloud Storage location, dataset partition, and corresponding RG or NRG label. The final imported dataset contained all 9,540 labeled images.

Model development was performed in the us-central1 region using the web-based Google Cloud Console. Model development was performed using the Google Cloud Vertex AI AutoML service available during the study period in August 2026. All investigator-selectable settings described in this study were retained as specified above; platform-controlled architecture selection, optimization procedures, and other internal settings were not accessible to the investigators because they are proprietary components of Vertex AI AutoML. AutoML was selected as the model-training method. Google Cloud Vertex AI automatically performed model-development processes including feature extraction, architecture selection, optimization, and hyperparameter tuning within its proprietary machine-learning framework. The final neural-network architecture and complete internal optimization strategy were not disclosed to the investigators.

No custom convolutional neural network was written by the investigators. The investigators also did not manually select a pretrained architecture, implement transfer learning, define an optimization algorithm, or conduct investigator-directed hyperparameter tuning. Model training was performed using a maximum computational budget of eight node-hours in accordance with the Google Cloud Vertex AI image-classification workflow [24].

Model evaluation

Overall average precision was designated as the primary model-performance metric. Precision and recall at the default confidence threshold, class-specific average precision, precision-recall curves, confidence-threshold analysis, and confusion-matrix performance were evaluated as secondary descriptive measures. Google Cloud Vertex AI automatically generated classification metrics including average precision, precision, recall, precision-recall curves, performance across prediction-confidence thresholds, class-specific average precision, and a row-normalized confusion matrix. According to the Google Cloud Vertex AI classification-evaluation schema, overall average precision corresponds to the area under the precision-recall curve and is micro-averaged for the overall model evaluation. Precision at a specified confidence threshold represents the proportion of model-generated classification predictions that are correct, whereas recall represents the proportion of relevant ground-truth classifications correctly identified by the model.

Unless otherwise specified, model performance was evaluated at the platform default confidence threshold of 0.50. No post hoc confidence threshold was selected to maximize performance. Because RG represented the clinically relevant referral class, the proportion of RG images correctly classified was additionally interpreted as the RG true-positive classification rate, while the proportion of NRG images correctly classified represented the NRG true-negative classification rate within this binary classification task.

Endpoint deployment and prediction

Following completion of model training, the AutoML classifier was deployed to an online prediction endpoint within Google Cloud Vertex AI. Representative retinal fundus photographs were submitted through the graphical prediction interface to demonstrate the functionality of the deployed system. For each submitted image, the endpoint returned predicted confidence scores for the RG and NRG classes.

Representative correctly classified examples from both diagnostic categories were selected for visualization. These examples were included solely to demonstrate endpoint inference and were not used as an additional quantitative test set or as independent evidence of model performance.

Statistical analysis

The primary analysis was descriptive. Classification metrics were generated automatically by Google Cloud Vertex AI from the held-out internal test dataset. No additional hypothesis testing or inferential statistical comparisons were performed. The AutoML classifier was not directly compared with a custom convolutional neural network trained and evaluated using the same dataset. Accordingly, comparisons with previously published glaucoma-classification models are descriptive and should not be interpreted as evidence of statistical equivalence or superiority.

Ethical considerations

The study used a publicly available, de-identified retinal-image dataset and did not involve prospective patient recruitment, intervention, or direct interaction with human participants.

## Results

Dataset and model training

A total of 9,540 color retinal fundus photographs were included in the study, consisting of 4,770 RG images and 4,770 NRG images. The Vertex AI dataset contained 7,632 training images (80.0%), 954 validation images (10.0%), and 954 held-out internal test images (10.0%). Model training was completed in approximately 1 hour and 53 minutes using a maximum computational budget of eight node-hours (Table 1).

**Table 1:** Dataset characteristics, training parameters, and internal test performance of the Google Cloud Vertex AI AutoML glaucoma classifier Dataset composition, computational training budget, and classification performance of the Google Cloud Vertex AI AutoML model on the held-out internal test dataset. Performance metrics were generated by Vertex AI, with overall precision and recall reported at the default confidence threshold of 0.50. AutoML: automated machine learning; RG: referable glaucoma; NRG: non-referable glaucoma.

Metric	Value
Total images	9,540
Referable glaucoma images	4,770
Non-referable glaucoma images	4,770
Training images	7,632 (80.0%)
Validation images	954 (10.0%)
Internal test images	954 (10.0%)
Maximum training budget	8 node-hours
Overall average precision	0.974
Overall precision	92.50%
Overall recall	92.50%
Average precision, NRG	0.977
Average precision, RG	0.977
Correct NRG classification	94%
Correct RG classification	91%

Overall classification performance

The AutoML classifier demonstrated strong performance on the held-out internal test dataset, achieving an overall average precision of 0.974. At the default prediction-confidence threshold of 0.50, overall precision and recall were both 92.5%. Precision-recall analysis demonstrated consistently high precision across most of the recall range, with a decline occurring primarily at extreme operating thresholds (Figure 1).

**Figure 1:**
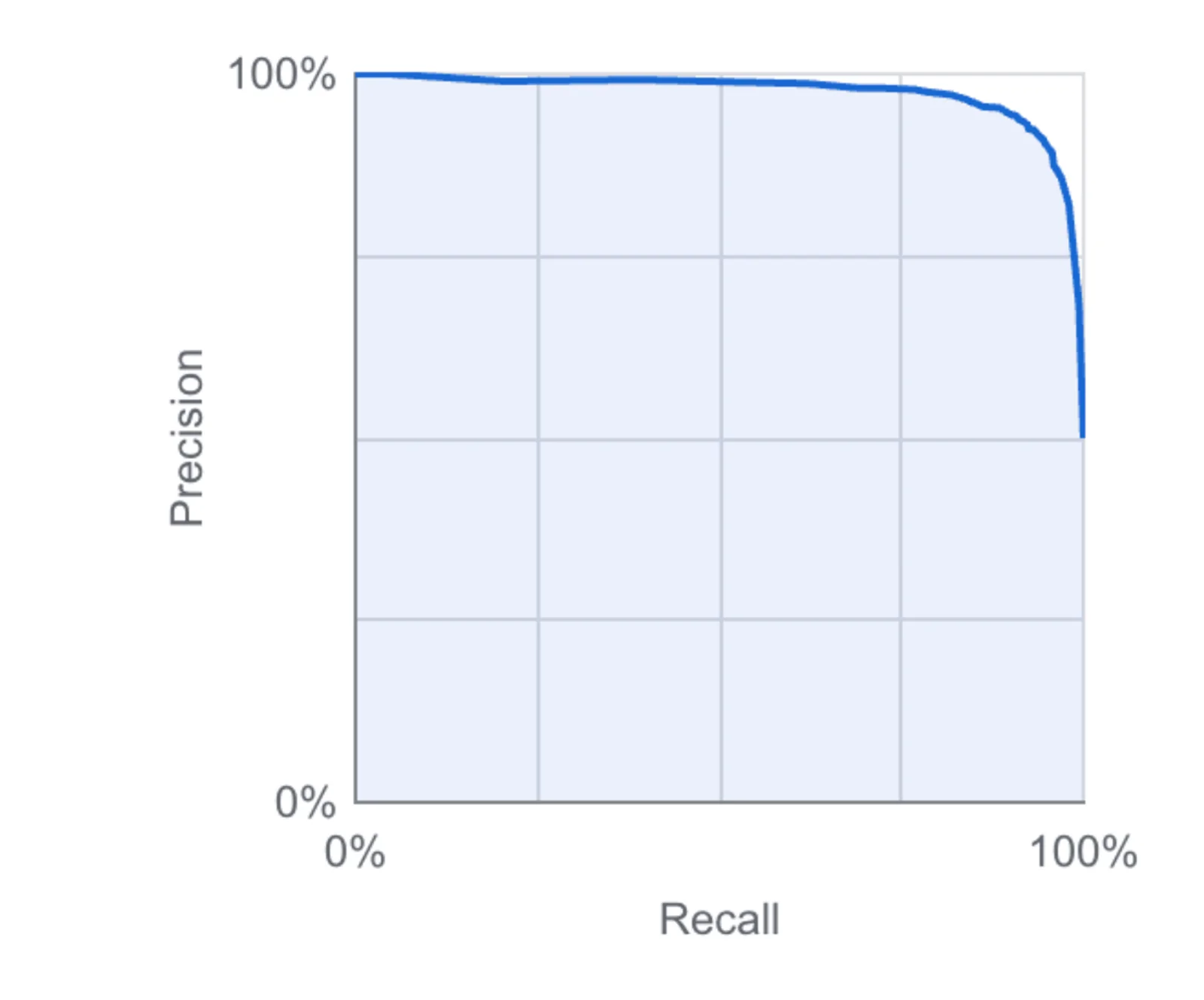
Precision-recall curve for the Google Cloud Vertex AI AutoML glaucoma classifier Precision-recall curve generated during evaluation of the Google Cloud Vertex AI AutoML glaucoma classifier on the held-out internal test dataset. The model maintained high precision across most of the recall range and achieved an overall average precision of 0.974. AutoML: automated machine learning.

Confidence-threshold analysis demonstrated the expected trade-off between precision and recall. As the prediction-confidence threshold increased, precision generally increased while recall decreased, with favorable performance maintained across a broad range of threshold values (Figure 2).

**Figure 2:**
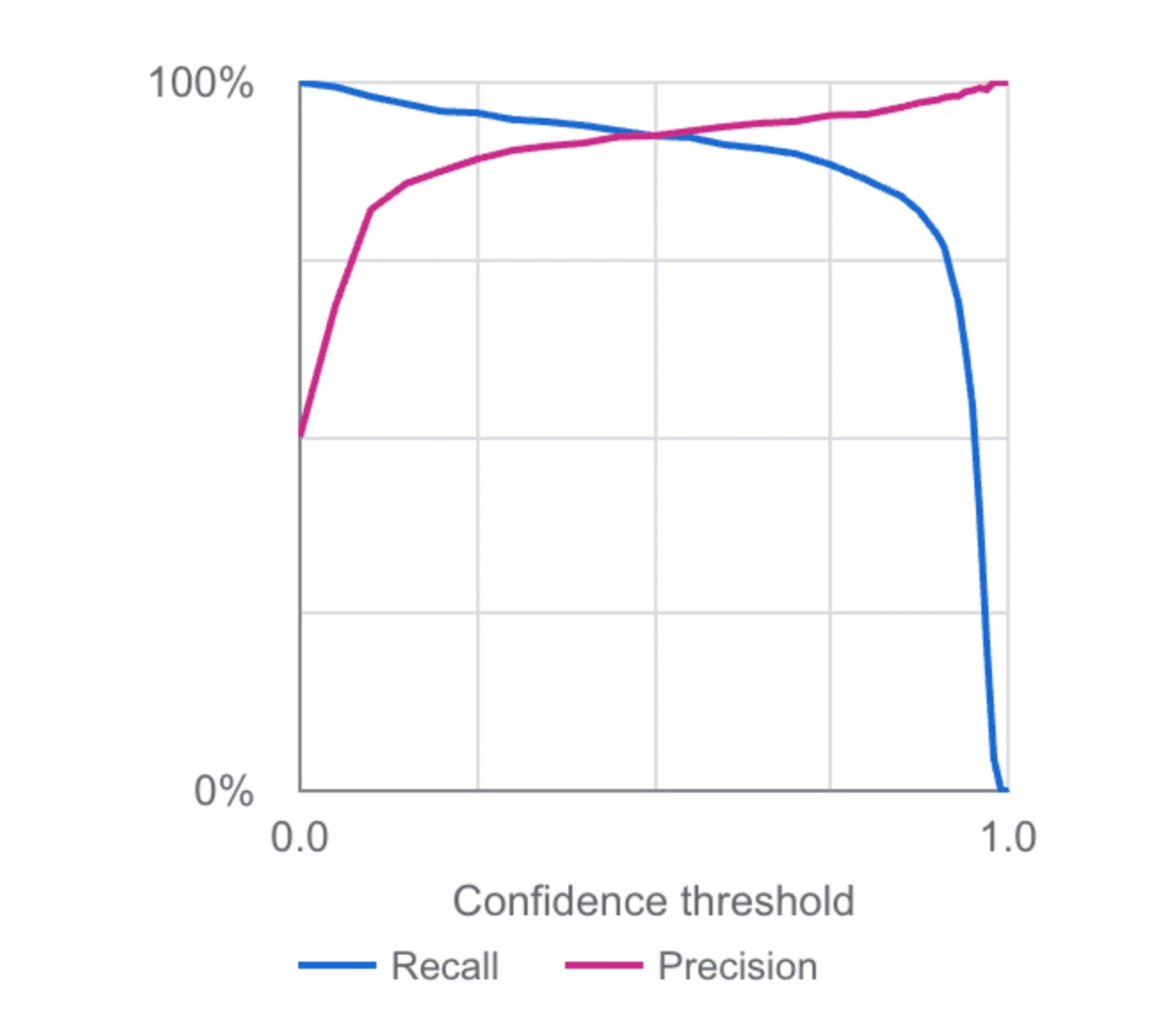
Precision and recall across prediction-confidence thresholds Precision and recall are plotted as functions of the model prediction-confidence threshold. Increasing the confidence threshold generally increased precision while decreasing recall, illustrating the expected trade-off between the two performance measures. AutoML: automated machine learning.

Class-specific performance

Class-specific average precision was 0.977 for both NRG and RG, demonstrating similar discriminatory performance across the two diagnostic categories. Row-normalized confusion matrix analysis showed that 94% of NRG images were correctly classified, while 6% were incorrectly classified as RG. Among RG images, 91% were correctly classified, while 9% were incorrectly classified as NRG. Thus, at the evaluated operating threshold, the model correctly identified 91% of RG images and correctly classified 94% of NRG images (Figure 3).

**Figure 3:**
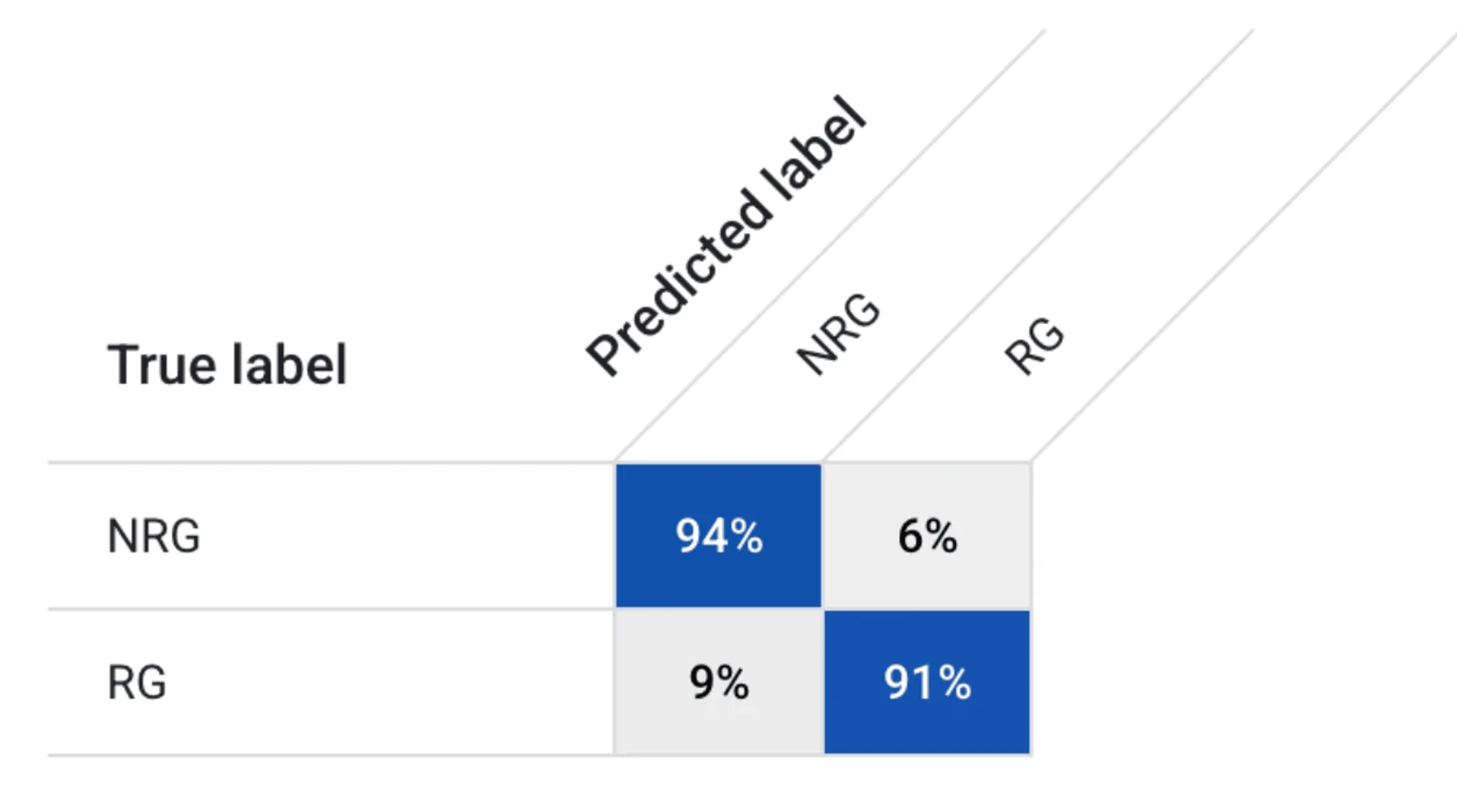
Row-normalized confusion matrix for the Google Cloud Vertex AI AutoML glaucoma classifier Row-normalized confusion matrix illustrating classification performance on the held-out internal test dataset. Rows represent the dataset-provided reference labels and columns represent model predictions. The model correctly classified 94% of non-referable glaucoma images, with 6% misclassified as referable glaucoma, and correctly classified 91% of referable glaucoma images, with 9% misclassified as non-referable glaucoma. AutoML: automated machine learning; RG: referable glaucoma; NRG: non-referable glaucoma.

Endpoint deployment

Following training, the AutoML classifier was successfully deployed to an online prediction endpoint within Google Cloud Vertex AI. Submission of representative retinal fundus photographs generated predictions for the RG and NRG classes together with corresponding confidence scores. Correctly classified examples from both diagnostic categories demonstrated the ability of the deployed cloud-based system to accept retinal images and return classification predictions through the graphical interface. These examples were selected solely to demonstrate endpoint functionality and were not used as an additional quantitative or independent test set (Figure 4).

**Figure 4:**
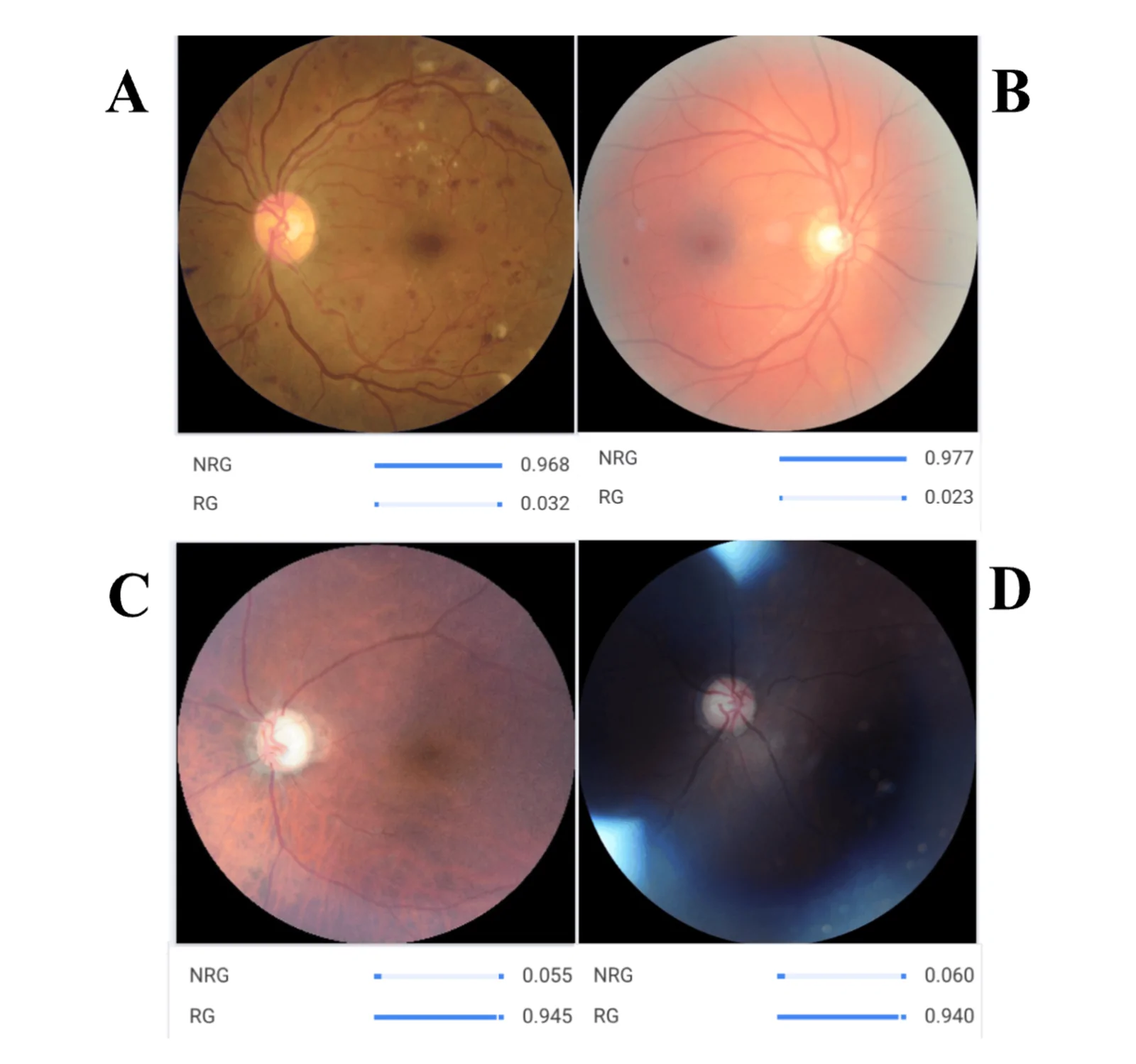
Representative predictions generated by the deployment of the Google Cloud Vertex AI AutoML glaucoma classification model Representative retinal fundus photographs correctly classified by the deployed Google Cloud Vertex AI AutoML endpoint. Panels A and B show images classified as non-referable glaucoma, while panels C and D show images classified as referable glaucoma. Corresponding prediction-confidence scores for both diagnostic categories are displayed beneath each image. These examples were selected to demonstrate endpoint functionality and do not represent an independent evaluation dataset. AutoML: automated machine learning; RG: referable glaucoma; NRG: non-referable glaucoma [23]. Image credit: Sourced from the EyePACS-AIROGS-light-V2 dataset [23], derived from the AIROGS training dataset, which is distributed under a Creative Commons Attribution-NonCommercial-NoDerivatives (CC BY-NC-ND) license [22].

## Discussion

In this proof-of-concept study, a commercially available AutoML platform generated a retinal fundus image classifier capable of distinguishing RG from NRG with strong performance on a held-out internal test dataset. The Google Cloud Vertex AI AutoML model achieved an overall average precision of 0.974 and overall precision and recall of 92.5% at the default confidence threshold of 0.50. Class-specific average precision was 0.977 for both RG and NRG, while the row-normalized confusion matrix demonstrated correct classification of 91% of RG images and 94% of NRG images. Importantly, the machine-learning portion of model development was completed through a graphical AutoML workflow without investigator-written machine-learning code, manual neural-network architecture construction, or investigator-directed hyperparameter optimization.

The principal contribution of this study is therefore not the development of a novel neural-network architecture or a claim of superiority over existing glaucoma classifiers. Numerous custom deep-learning systems have already demonstrated strong performance for identifying glaucomatous or referable optic nerve abnormalities from retinal fundus photographs [8-14]. Rather, the present study demonstrates that strong internal classification performance can be achieved using an accessible commercial AutoML platform that substantially reduces the technical expertise traditionally required for model development. This distinction is particularly relevant for clinicians and biomedical investigators who may have expertise in disease-specific research but limited experience with programming or neural-network engineering.

Previous investigations have similarly examined automated and code-free approaches to medical image classification. Faes et al. demonstrated that healthcare professionals without coding experience could use automated deep-learning tools to develop classifiers across multiple medical imaging tasks [16]. Korot et al. subsequently evaluated code-free deep-learning approaches across multiple imaging modalities and demonstrated their potential to lower technical barriers to AI development while emphasizing the continued importance of rigorous dataset design and validation [17]. More recent investigations have continued to evaluate AutoML approaches for medical image classification and their potential role in making AI development more accessible to nontechnical investigators [18,19]. The present study extends this literature by applying a fully graphical Google Cloud Vertex AI AutoML workflow specifically to binary classification of RG from NRG using retinal fundus photography.

Although the performance observed in this study falls within the broad range reported for several previously published deep-learning approaches to glaucoma classification, direct numerical comparisons should be interpreted cautiously [8-14]. Published studies differ substantially in dataset composition, preprocessing methods, reference standards, glaucoma definitions, class prevalence, image-acquisition systems, test populations, and reported performance metrics. Furthermore, the present study did not perform a head-to-head comparison between AutoML and a custom convolutional neural network trained and evaluated using identical data. The current results therefore do not establish equivalence or superiority of AutoML compared with investigator-designed deep-learning architectures.

The confusion matrix provides additional context regarding clinically relevant classification errors. The model correctly classified 91% of RG images, whereas 9% of RG images were classified as NRG. In a screening setting, these false-negative classifications would be particularly important because individuals with potentially referable disease could fail to receive appropriate ophthalmologic evaluation. Conversely, 6% of NRG images were classified as RG, which could result in unnecessary referrals and additional resource utilization. The optimal balance between sensitivity to referable disease and avoidance of unnecessary referral depends on the intended clinical setting and disease prevalence. In the present study, the platform's default confidence threshold of 0.50 was evaluated rather than a prospectively defined threshold optimized for clinical screening.

The intentionally balanced composition of the study dataset also requires consideration when interpreting the results. Equal numbers of RG and NRG images were included, whereas the prevalence of RG in real-world screening populations would generally be considerably lower. Measures such as precision and predictive values are influenced by disease prevalence; consequently, performance observed in a balanced experimental dataset cannot be assumed to represent expected performance in a routine screening population. Evaluation in cohorts that reflect the prevalence, demographic characteristics, disease spectrum, and image-quality distribution of the intended clinical population is necessary before clinical utility can be estimated.

An important advantage of the AutoML approach is its reduction of the technical burden associated with conventional deep-learning development. Traditional machine-learning workflows may require decisions regarding neural-network architecture, transfer learning, optimization algorithms, learning rates, regularization, data augmentation, and numerous other hyperparameters. In the present study, these processes were largely abstracted by the AutoML platform. This may allow clinicians and biomedical researchers to rapidly evaluate whether a medical imaging classification problem contains sufficient discriminatory information to justify further development without first acquiring extensive programming expertise.

However, code-free model development should not be interpreted as eliminating the need for methodological expertise. The quality and clinical relevance of an AI system remain dependent on appropriate study design, representative datasets, reliable reference standards, prevention of data leakage, selection and interpretation of performance metrics, assessment of bias, external validation, and evaluation within the intended clinical environment. Contemporary reporting recommendations for medical AI similarly emphasize transparent reporting of dataset construction, model evaluation, reference standards, and validation procedures regardless of whether the underlying model is developed manually or through an automated platform [20,21].

The proprietary nature of Google Cloud Vertex AI also introduces an important trade-off between accessibility and transparency. Although the platform simplifies model development, the final neural-network architecture and complete internal optimization strategy are not available to investigators. This limits mechanistic transparency and makes exact architectural replication more difficult than with an openly specified custom model. In addition, commercial AutoML platforms may change over time as underlying algorithms and services are updated. Consequently, reproducibility depends not only on the dataset and investigator-defined workflow but also on the version and functionality of the commercial platform available at the time of training.

A further consideration is how a code-free glaucoma classifier might ultimately integrate into clinical workflows. The present study evaluated technical feasibility and internal classification performance rather than clinical implementation, and the model was not tested prospectively within an ophthalmology or screening environment. In practice, an automated fundus-image classifier could potentially function as a decision-support or triage tool by identifying images with features suggestive of RG and prioritizing those patients for ophthalmologic review. Such use would require integration with existing imaging and electronic health record workflows, clearly defined referral thresholds, and mechanisms that allow clinicians to interpret predictions in the context of other clinical information. Clinician trust will also be important, particularly because the proprietary AutoML architecture limits direct inspection of the model and the present study did not evaluate explainability or clinician interaction with model outputs. Future studies should therefore assess not only external diagnostic performance but also how automated predictions influence clinician decision-making, referral behavior, workflow efficiency, diagnostic confidence, and patient outcomes. Prospective human-in-the-loop studies will be necessary to determine whether such systems improve clinical decision-making without introducing inappropriate reliance on automated predictions.

Several limitations of the present study should be acknowledged. First, model performance was evaluated using a held-out test subset originating from the same overall derivative dataset used for model development. Although these images were not used for model fitting, this represents internal testing rather than external validation. In addition, because partitioning was performed at the image level rather than at the patient or eye level, images from the same participant or paired eyes could potentially have been present in different partitions. This introduces a potential risk of information leakage and may have influenced the reported internal performance estimates. Future studies should use patient-level partitioning, when participant identifiers are available, to ensure complete independence between model-development and evaluation datasets. Alternative validation approaches were considered; however, additional resampling strategies such as k-fold cross-validation were not pursued because the study preserved the predefined training, validation, and test partitions throughout model development, and such approaches would not address the more important question of generalizability to an independent population. External validation was not performed because this proof-of-concept investigation was designed around a single publicly available benchmark dataset, and incorporation of a separate cohort would require an independent dataset with compatible RG and NRG reference labels, preprocessing, and image characteristics, which was beyond the scope of the present study. Consequently, generalizability to images acquired from different patient populations, healthcare systems, camera manufacturers, acquisition protocols, and image-quality distributions remains unknown. Future studies should externally validate the model using independent datasets from different institutions and imaging systems before claims regarding clinical generalizability or implementation can be made.

Second, the study relied on the RG and NRG labels supplied with the publicly available dataset. The investigators did not independently adjudicate or relabel the retinal images. Any uncertainty or misclassification within the source labels could therefore affect both model development and performance evaluation. Third, the retinal photographs used in this investigation were obtained from a preprocessed derivative of the original AIROGS dataset rather than directly from an unprocessed clinical imaging workflow. According to the dataset documentation, images had undergone standardized cropping, centering, resizing, and masking before public distribution [23]. The reported results therefore reflect the combination of this preprocessing pipeline and the subsequent AutoML model and cannot establish how the same workflow would perform on unprocessed fundus photographs obtained directly from clinical practice.

Fourth, the study did not prospectively compare the model with ophthalmologists, glaucoma specialists, or another AI classifier. No conclusions regarding equivalence or superiority to clinician interpretation or custom deep-learning architectures can therefore be made. Fifth, the present analysis was descriptive, and additional inferential statistical testing and confidence-interval estimation were not performed. Small numerical differences between performance measures should consequently not be interpreted as statistically meaningful.

Sixth, this study evaluated binary image classification rather than comprehensive glaucoma diagnosis. Clinical diagnosis and management of glaucoma may incorporate intraocular pressure, optic nerve examination, optical coherence tomography, visual-field testing, gonioscopy, patient-specific risk factors, previous examinations, and longitudinal change. A fundus image classifier should therefore be considered a potential component of a screening or clinical decision-support workflow rather than a replacement for comprehensive ophthalmologic evaluation.

Finally, model interpretability was not directly evaluated. Explainable AI approaches, including saliency-based and class-activation techniques, may help identify the retinal regions contributing to model predictions and facilitate analysis of both correct and incorrect classifications [15]. Such approaches may be particularly useful for AutoML systems in which the underlying neural-network architecture is proprietary and not directly accessible to investigators.

Future investigations should externally validate AutoML-generated glaucoma classifiers using independent datasets from different institutions, populations, and imaging devices. Prospective evaluation in screening populations with realistic disease prevalence would allow assessment of clinically meaningful measures such as predictive values and referral performance. Future studies should also directly compare AutoML-generated models with conventional investigator-designed architectures trained and tested on identical datasets, evaluate calibration and clinically prespecified operating thresholds, assess performance across relevant demographic and clinical subgroups, and incorporate explainability methods when technically feasible. These investigations would help clarify the appropriate role of code-free AutoML platforms along the continuum from exploratory model development to clinically validated artificial intelligence.

## Conclusions

This proof-of-concept study demonstrates that Google Cloud Vertex AI AutoML can generate a high-performing retinal fundus image classifier for distinguishing RG from NRG through a graphical workflow that does not require investigator-written machine-learning code or manual neural-network architecture design. The model demonstrated high overall performance, with similar class-specific performance for both diagnostic categories.

These findings support the feasibility of commercial AutoML platforms as accessible tools for ophthalmic image-classification research, particularly for clinicians and biomedical investigators without extensive programming or machine-learning expertise. However, the results represent internal testing on a single, preprocessed derivative dataset and should not be interpreted as evidence of clinical effectiveness, superiority to conventional deep-learning approaches, or generalizability to real-world glaucoma screening populations. External validation using independent institutions, patient populations, imaging systems, and clinically representative disease prevalence is necessary before clinical implementation can be considered. Future studies should also evaluate prospective screening performance, clinically defined decision thresholds, subgroup performance, model calibration, and direct comparisons with conventional deep-learning architectures.
